# Consequence of the introduction of routine FCH PET/CT imaging for patients with prostate cancer: a dual centre survey

**DOI:** 10.2478/raon-2013-0049

**Published:** 2014-01-22

**Authors:** Marina Hodolic, Laure Michaud, Virginie Huchet, Sona Balogova, Valérie Nataf, Khaldoun Kerrou, Marika Vereb, Jure Fettich, Jean-Noël Talbot

**Affiliations:** 1 Department for nuclear medicine, University Medical Centre, Ljubljana, Slovenia; 2 Médecine nucléaire, Hôpital Tenon, AP-HP, Paris, France; 3 Université Pierre et Marie Curie, Paris, France; 4 Comenius University, Bratislava, Slovakia; 5 Radiopharmacie, Hôpital Tenon, AP-HP, Paris, France; 6 Nuklearmedizin, Kassel Klinikum, Kassel, Germany

**Keywords:** prostate cancer, PET/CT, fluorocholine (FCH), fluoride(18F), bone scintigraphy, indication of imaging

## Abstract

**Background:**

Fluorocholine(18F) (FCH) was introduced at the beginning of April 2010 in France, Slovenia and three other EU member states for the localisation of bone metastases of prostate cancer with PET. The aim of the study was to compare the evolution of diagnostic imaging in patients with prostate cancer using a new radiopharmaceutical FCH, observed in France and in Slovenia, and to quantify the consequence of the results of new imaging modality on the detection rate of abnormal metastases and recurrences of prostate cancer.

**Patients and methods:**

In two centres (France/Slovenia), a survey of the number of nuclear medicine examinations in patients with prostate cancer was performed, covering 5 quarters of the year since the introduction of FCH. For each examination, the clinical and biological circumstances were recorded, as well as the detection of bone or soft tissue foci.

**Results:**

Six hundred and eighty-eight nuclear medicine examinations were performed impatients with prostate cancer. Nuclear medicine examinations were performed for therapy monitoring and follow-up in 23% of cases. The number of FCH PET/CT grew rapidly between the 1^st^ and 5^th^ period of the observation (+220%), while the number of bone scintigraphies (BS) and fluoride(18F) PET/CTs decreased (−42% and −23% respectively). Fluorodeoxyglucose(18F) (FDG) PET/CT remained limited to few cases of castrate-resistant or metastatic prostate cancer in Paris. The proportion of negative results was significantly lower with FCH PET/CT (14%) than with BS (49%) or fluoride(18F) PET/CT (54%). For bone metastases, the detection rate was similar, but FCH PET/CT was performed on average at lower prostate-specific antigen (PSA) levels and was less frequently doubtful (4% *vs*. 28% for BS). FCH PET/CT also showed foci in prostatic bed (53% of cases) or in soft tissue (35% of cases).

**Conclusions:**

A rapid development of FCH PET/CT was observed in both centres and led to a higher detection rate of prostate cancer lesions.

## Introduction

Among nuclear medicine diagnostic procedures, four are currently routinely used in patients with prostate cancer: bone scintigraphy (BS); fluoride(18F) PET/CT; fluorodeoxyglucose(18F) (FDG) PET/CT; fluorocholine(18F) (FCH) PET/CT. There is a clear difference between BS and fluoride(18F) PET/CT which are suited only for the detection of bone metastasis, and FCH and FDG PET/CT which can also detect primary tumour and soft tissue lesions.

In France bisphosphonates (99mTc) were registered in 1992, FDG in 1998, fluoride(18F) in 2008 and FCH (IasoCholine, IASON, Graz, Austria) become available in 2010. We published a survey that showed the shift in the prescription of nuclear medicine imaging favouring FCH PET/CT at Hospital Tenon in Paris, after its registration.^1^ All those radiopharmaceuticals have marketing authorisation and are available for the routine use also in five other EU member states: Austria, France, Germany, Poland and Slovenia.

The aim of the present article is to compare the evolution of diagnostic imaging in patients with prostate cancer using a new radiopharmaceutical FCH observed in France (Paris, Hospital Tenon), with evolution of corresponding imaging in an Central European country (Slovenia, University Medical Centre Ljubljana), and to quantify the consequence of the results of new imaging modality on the detection rate of metastases and recurrence of prostate cancer.

## Patients and methods

### Centres and data collection

Covering a period from 2^nd^ April 2010 to 1^st^ July 2011 (both included), *i.e*. 5 quarters, in two nuclear medicine centres (Table 1), the data base includes: the age of the patient; the type of nuclear medicine examination; the indication: initial staging, follow-up during or just after treatment, restaging (of a known recurrence), or occult biological recurrence; the total number of previous nuclear medicine examinations; a quotation of the result of each examination performed by two independent nuclear medicine specialists experienced in the 4 types of examinations (S.B. and M.V.) from the images and the report: negative, doubt for bone metastasis, highly evocative of bone metastasis, focus (foci) in the prostatic bed, doubtful soft tissue focus or foci, highly evocative of soft tissue metastasis. For one abnormal examination, several categories could thus be quoted.

In some cases, there were also available: the serum prostate-specific antigen (PSA) levels (ng/mL) at the time of the nuclear medicine examination (502 cases); the initial Gleason score (361 cases); and both: PSA level and Gleason score (299 cases). The investigators followed recommendations of the Helsinki Declaration. The study protocol was approved by the ethic committees of both participating centres.

### Data processing and statistics

The number of examinations performed in patients with prostate cancer disease was determined for each of the 4 nuclear medicine examinations, for each quarter and for each centre. The comparison of the 1^st^ and 5^th^ quarter is of particular interest; since they correspond to the same months (April to June) of 2 consecutive years (2010 and 2011) avoiding the consequences of an influence of the season (feasts, vacations…) on the number of prescribed nuclear medicine examinations. Those proportions were compared using chi-square test.

Differences in age, serum PSA levels, and Gleason score between the patients, according to the prescribed nuclear medicine examination, were tested by Kruskal-Wallis test. In case only two alternatives exist, the Mann-Whitney test was used. In patients who benefited from several nuclear medicine examinations during the survey period, the number, the type and the sequence of the prescribed examinations were reported and analysed.

## Results

### Evolution of the prescription of nuclear medicine examinations

Overall, 688 nuclear medicine examinations were performed in 577 patients with prostate cancer during the survey period. In Paris, the most frequently prescribed examination was fluoride(18F) PET/CT (147 cases), very close to FCH PET/CT (145 cases), then BS (103 cases) and finally FDG PET/CT (34 cases) mostly prescribed in case of advanced cancer, with frequent repetition in the same patient during the survey period. During the same period of time, a total of 951 BS and 3896 whole-body PET/CT were performed in this centre: prostate cancer was the indication of 103 out of 951 BS (10.8%) and in 326 out of 3896 PET/CT (8.4%). The ratio of BS in patients with prostate cancer disease decreased from 15% in the 1^st^ quarter to 6% in the 5^th^ quarter. Conversely, PET/CT examinations in patients with prostate cancer increased from 6% in the 1^st^ quarter to 9% in the 5^th^ quarter (Table 2).

In Ljubljana, fluoride(18F) PET/CT was not available and the most frequently prescribed examination in patients with prostate cancer was FCH PET/CT (230 cases), BS (29 cases) and FDG PET/CT (1 case). During the same period of time, a total of 1757 BS and 2069 PET/CT were performed in this centre: prostate cancer was the indication in 29 out of 1757 BS (1.7%) and in 213 out of 2069 PET/CT (10.3%) (Table 3).

Tables 2 and 3 illustrate the evolution of the prescribed nuclear medicine examination during the 5 successive quarters in each centre. The chi-square test is very significant (p <<0.001): there was an increase in the proportion of FCH PET/CT with time, and a decrease in the proportion of BS in both centres and also of fluoride(18F) PET/CT in Paris.

### Multiple nuclear medicine examinations in the same patient during the survey period

Sixty-seven patients had multiple nuclear medicine examinations, ranging between 2 and 11 examinations per patient (in Ljubljana, a maximum of 2 examinations were performed for one single patient during the 5 quarters).

The main prescription patterns were:

- 2 or 3 examinations of the same type in the mentioned interval: BS in 2 patients, fluoride(18F) PET/CT in 3 patients, FDG PET/CT in 1 patient, FCH PET/CT in 8 patients.
- 2 examinations of different type within less than one month: fluoride(18F) PET/CT and FCH PET/CT in 3 patients, fluoride(18F) PET/CT and FDG PET/CT in 1 patient and, most frequently in Ljubljana, BS and then FCH PET/CT in 19 patients, the reverse in only 1 patient.
- a shift to another type of examination prescribed on the next visit, after several months: fluoride(18F) PET/CT to FCH PET/CT in 4 patients, fluoride(18F) PET/CT to FDG PET/CT in 1 patient, FCH PET/CT to FDG PET/CT in 2 patients, BS to FCH PET/CT in 13 patients, BS to fluoride(18F) PET/CT in 1 patient.
- at least 3 different types of examination repeated within the survey period in 8 patients.

### Clinical context

Mean age of patients included in the study was 68.4 years (range 45–97 years). As expected, there was a significant difference in age according to the indication of the nuclear medicine examination. The patients being referred for initial staging being younger (mean age 67.2 years) and the patients referred for occult recurrence older (mean age 69.8 years) (p=0.01).

The choice of the nuclear medicine examination was in relation with the indication (p <<0.001) (Table 4). BS and fluoride(18F) PET/CT were performed more frequently for the initial staging, while FCH PET/CT was performed in almost half of the cases for an occult recurrence. As already mentioned, FDG PET/CT was mostly used for therapy follow-up or restaging of advanced castration-resistant forms.

In accordance, the number of previous nuclear medicine examinations performed in the patient and recorded by the centre was significantly greater when FDG PET/CT was requested. The mean number of previous examinations was 0.4 when BS was prescribed, 0.8 when fluoride(18F) PET/CT was prescribed, 0.7 when FCH PET/CT was prescribed *vs*. 4.8 for FDG PET/CT.

### Biological context

A significant relation was observed between the PSA serum levels and the type of the prescribed nuclear medicine examination (p<<0.001). The mean PSA level was 26 ng/ml when FCH PET/CT was prescribed, 24 ng/ml in case of FDG PET/CT, 74 ng/ml in case of fluoride(18F) PET/CT and 175 ng/ml when the patient was referred for BS. The difference in PSA levels according to the indication did not reach the level of significance. The initial Gleason score of the patients referred for FDG PET/CT (mean 8.4) was significantly greater than that of patients referred for all other examinations (mean 7.4 for BS, 7.6 for fluoride(18F) PET/CT and 7.3 for FCH PET/CT); it was also higher in patients referred for the treatment follow-up (mean 7.8) or restaging (mean 7.8) than in case of initial staging (mean 7.4) or occult recurrence (mean 7.2).

### Nuclear medicine examination report: normal, positive, doubtful

Table 5 illustrates the result of the report summarized on an examination-based manner, according to the findings and the type of nuclear medicine examination. BS or fluoride(18F) PET/CT was interpreted as normal in around one half of cases, without difference in the distribution of the “positive” and “doubtful” conclusions (on a per-examination level) between those two modalities. In one patient, a large lymph node took-up fluoride(18F).

FDG PET/CT results favoured bone metastases in 85% of patients and less frequently reported soft tissue foci evocative of malignancy. This does not mean that FDG is better than fluoride(18F) or FCH to detect bone metastases but, in accordance with previous results, that FDG PET/CT was prescribed in patients with advanced forms of the disease, mostly castration-resistant and metastatic to the skeleton, for restaging or chemotherapy monitoring.

**FIGURE 1.**
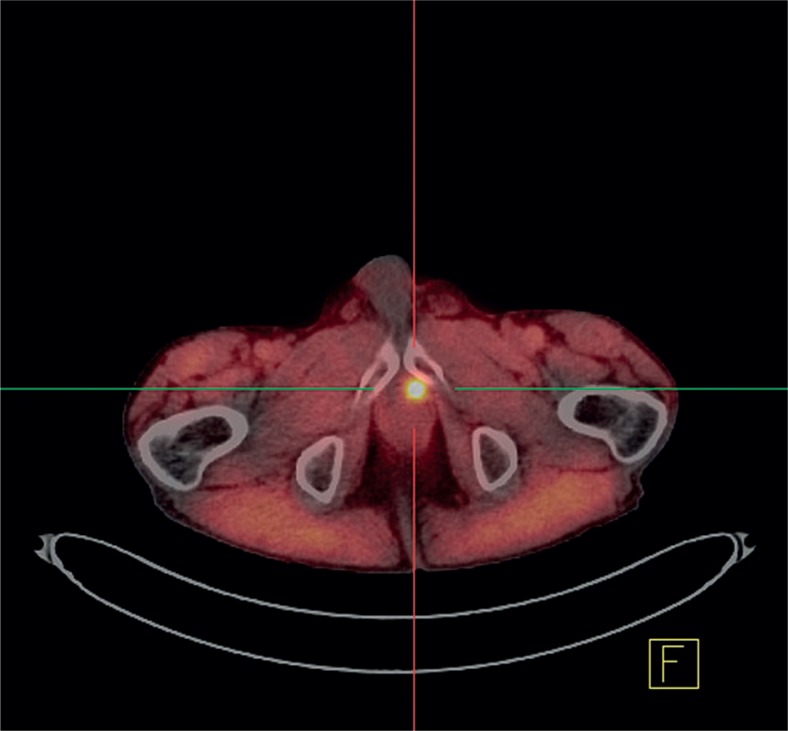
FCH PET/CT: Local recurrence of prostate cancer after radical prostatectomy (Gleason score 8, [PSA] 0.2 ng/ml).

**FIGURE 2.**
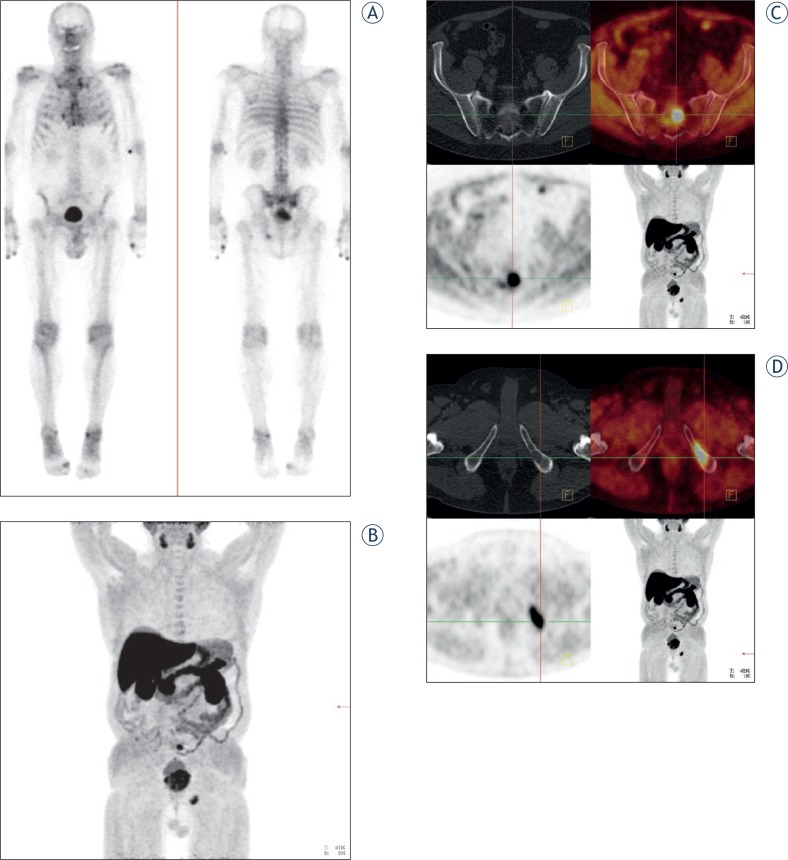
**A** Bone scintigraphy: Pathological tracer uptake in the left os ischii in a patient with prostate cancer (Gleason score 7; [PSA] 30 ng/ml) – initial staging. **B** FCH PET/CT (MIP image): Pathological FCH uptake in the sacral region as well as in the left os ischii in the same patient. **C** FCH PET/CT axial slice: Pathological FCH uptake in the sacral region. **D** FCH PET/CT axial slice: Pathological FCH uptake in the left os ischii.

FCH PET/CT was abnormal in 86% of patients and doubtful in a small minority of the examinations. It showed the primary tumour or a local recurrence in the prostatic bed in about half of the patients (Figure 1), foci suspicious for soft tissue malignancy in about one third, and also foci evocative of bone metastases, in a proportion of patients (23%) similar to that of BS or fluoride(18F) PET/CT (p>0.9), but with significantly less doubtful cases (p< 0.001) (Figure 2).

Reporting can also be analysed according to the indication of nuclear medicine imaging. As already mentioned, FDG PET/CT was most frequently prescribed for restaging and follow-up of response to treatment, in patients whose advanced prostate cancer was already known to be metastatic. In this context, the metastatic spread, in particular to the skeleton, was visible on FDG PET/CT in 97% of cases.

In the search for bone metastases, no difference in the frequency of detection was found according to the indication with BS while fluoride(18F) PET/CT and FCH PET/CT showed more frequently suspicious bone foci when performed for restaging or treatment follow-up, probably in relation with already known metastatic dissemination in those patients. The frequency of detection of suspicious bone foci in patients with a Gleason score less than or equal to 7, was 5% for BS, 8% for fluoride(18F) and 12% for FCH, in patients with a Gleason score greater than or equal to 8, the corresponding values were 35%, 23%, and 32% (the difference was significant for BS and FCH PET/CT).

Searching for malignant deposits in soft tissue, FCH PET/CT was more frequently positive in patients referred for restaging or occult recurrence than at initial staging (p<0.01). The detection rate of suspicious soft tissue foci was 27% in patients with a Gleason score less than or equal to 7, *vs*. 31% in patients with a Gleason score greater than or equal to 8 (p > 0.6).

In our centres, some examinations were performed at initial staging in patients who did not fulfil accepted criteria to refer patients at initial staging to nuclear medicine imaging, *i.e*. PSA levels less than or equal to 10 ng/ml and Gleason score less than 8. They corresponded to 36 of the 132 examinations (27%) performed for initial staging in patients whose PSA serum levels and Gleason score were mentioned on the prescription.

In case of biochemical recurrence following prostatectomy, the NCCN Guidelines mention a potential indication for BS without precise target PSA value. NCCN Guidelines also recommends BS in case of post-irradiation recurrence in patients who are considered candidates for local therapy, with PSA less than 10 ng/ml among other criteria.^2^ In our survey, 133 examinations were performed for restaging or detection of occult recurrence in patients with PSA levels less than 10 ng/ml. Foci suspicious to correspond to malignant tissue out of the prostatic bed were reported in 1 out of 10 BS, 2 out of 15 fluoride(18F) PET/CT, 1 out of 1 FDG PET/CT and 50 out of 117 FCH PET/CT. This very significant superiority of FCH PET/CT over bone nuclear medicine imaging (p<0.01) is due to its ability to detect soft tissue lesions as well as bone lesions. In those patients, FCH PET/CT also showed foci in the prostatic bed suspicious for local recurrence in 41 cases (35%). In this context of recurrent disease, FCH PET/CT was prescribed in 23 patients with PSA levels < 2 ng/ml and initial Gleason score less than or equal to 7: its detection rate (including local recurrence) was still 35%.

## Discussion

As its first result, this dual centre study confirms, in two independent nuclear medicine centres, the rapid rise in the demand for FCH PET/CT, as soon as FCH was registered.^1^ At the same time, there was a marked decline in the prescription of BS in patients with prostate cancer. This shift was associated with a rise of the total number of prostate cancer patients referred for nuclear medicine examinations. The transfer of prescription to FCH PET/CT was more progressive in Paris than in Ljubljana. Bone PET/CT with fluoride(18F) has been available in Paris for one year and a half when FCH was registered, yielding images with PET quality and a superior resolution as compared to BS or bone single photon emission computed tomography (SPECT). Even for the most informed prescribers, the introduction of FCH meant two successive shifts in a limited period of time. Another reason can be the relation with the environment. The Paris area has 11.7 millions inhabitants and 42 nuclear medicine centres, 20 of which are equipped with PET/CT, which means a rather large resource for the prescriber, while Slovenia has 2 million inhabitants, 7 nuclear medicine centres and 2 PET/CT centres (FCH is being performed in one), which probably enables a more rapid diffusion of new PET imaging modalities.

The other aim of this survey was to record the detection of abnormal foci by the available nuclear medicine examinations, but not to compare their performance according to a standard of truth. Actually most patients only had one examination, and head to head comparison of results, according to the imaging modality, is not possible. Nevertheless, it is of importance to check how this concordant and rapid evolution in Paris and Ljubljana is based on evidence and matches results obtained in other centres.

The initial shift from BS to bone PET/CT with fluoride(18F), which has been observed in Paris^1^, is in agreement with the results of the comparative study of Even-Sapir *et al*., in 44 patients with a high-risk prostate cancer.^3^ Fluoride(18F) PET/CT was statistically more sensitive and more specific than planar BS or bone SPECT (p < 0.05). In our survey, the majority of fluoride(18F) PET/CT has been performed to search for bone metastases at initial staging, to profit from the better sensitivity. The advantage of fluoride(18F) PET/CT over BS and bone SPECT was not so obvious when examining reporting of examinations (Table 5) because fluoride(18F) PET/CT mostly results in the detection of a greater number of bone lesions as compared to BS, while the analysis of our results was based on a per-patient level rather than a per-lesion level. The further shift from fluoride(18F) to FCH as the PET/CT tracer to detect bone metastases is evaluated by the comparative studies from the team in Linz in co-operation with our team in Paris.^4^ In this study, there was no significant difference in sensitivity between the two PET tracers, but FCH was significantly more specific on a lesion-based analysis. In the present survey, the use of FCH instead of BS, bone SPECT or fluoride(18F) PET/CT resulted in a similar proportion of examinations interpreted as positive for bone metastases, and in a decrease in the frequency of doubtful reports: in contrast with bisphosponate (99mTc) or fluoride(18F), FCH is not taken-up by non-inflammatory degenerative changes in the skeleton and its interpretation is more straightforward.^5^

Concerning the detection of lesions in the prostatic bed, locoregional lymph nodes and distant soft tissue, FCH is in competition with FDG. FDG has a low diagnostic performance in the general population of prostate cancer patients, but may be of interest in case of aggressive or castration-resistant prostate cancer. The analysis of the US national oncologic PET registry for the first 2 years of data by Hillner *et al*. revealed that, from 40,863 PET scans, prostate cancer was the most frequent indication corresponding to 5,309 FDG examinations, with change in management in 35% of cases.^6^ However, also FCH is taken-up by androgen-independent prostate cancer, as showed as early as 2002 by Price *et al*. in 9 patients^7^ and confirmed recently by Mc Carthy *et al*., in 26 patients.^8^ In the present survey, FDG PET/CT was performed, in Paris only, in a very limited number of patients with a high Gleason score, to restage a known recurrence and to monitor therapy of metastatic forms, when re-staging FCH PET/CT was positive. The prescription of FDG PET/CT in prostate cancer was not increasing with time, in contrast with that of FCH PET/CT.

The utility of FCH PET/CT to detect recurrent prostate cancer has been demonstrated by several teams since 2005^9^, a special attention being paid to the rate of positive examinations according to PSA levels^10^–^14^ or PSA doubling time or velocity^15^,^16^, or initial Gleason score.^11^ In our survey, 51% of the FCH PET/CT was performed for restaging a known recurrence or localising an occult biological recurrence. The reported relation between the frequency of positivity and PSA levels and the initial Gleason score has been observed in our series. However, FCH PET/CT detected suspicious foci in 35% of patients with PSA levels < 2 ng/mL and initial Gleason score less than or equal to 7. According to Pelosi *et al*., its detection rate was still 20% when PSA levels were < 1 ng/ml.^13^ Even though FCH is for the moment only registered for the detection of bone metastases, it is also able to detect local recurrences (Figure 1) and locoregional lymph node metastases.

The utility of FCH PET/CT in the initial staging of prostate cancer has been addressed by Beheshti *et al*.^17^ In this context, FCH PET/CT has limited value in the detection of malignant lymph nodes especially when smaller than 5 mm, but it led to changes in the therapeutic management of 20% of prostate cancer patients at a high risk for extracapsular disease, suggesting that it will be helpful in triaging care of this type of patient cohort. Patient-based sensitivity was 73% and specificity 88% in 210 intermediate or high-risk patients showing FCH PET/CT to be effective to detect N+ patients.^18^ In our survey, 26% of the FCH PET/CT were performed at initial staging and not only visualised the primary cancer but also detected suspicious foci in soft tissue or in the skeleton in 31% of patients (Table 5). A recent study confirmed that, at staging, when PSA levels (> 20 ng/l) and/or Gleason score (8–10) are high, both FCH and fluoride(18F) PET/CT were effective and impacted on the treatment plan for 20% of the patients.^19^ Should the classical criteria recommended for performing BS, *i.e*. PSA levels greater than or equal to 10 ng/ml or Gleason score of at least 8, also apply to PET/CT?^20^ In our series, its yield was actually rather low when those criteria were not met: 2 cases of extraprostatic foci in 14 examinations. In the survey of Lavery *et al*. “overuse” of BS in patients who did not fulfil somewhat less though criteria (a Gleason score of 7 was accepted for indication) occurred in 241 of 667 preoperative imaging examinations (36%); BS were read as positive in 21 cases (9%) which all corresponded to false-positive results.^21^ When the criteria used by Lavery *et al*.^21^ were applied to examinations performed in our series at initial staging, only 20% of BS, 15% of fluoride(18F) PET/CT and 7% FCH PET/CT should not have been performed, but their yield was even lower than with “classical” criteria: positivity was reported in none of the BS, 1 fluoride(18F) PET/CT and 1 FCH PET/CT. Thus, in staging prostate cancer, the overuse of nuclear medicine imaging was less frequent in Paris and Ljubljana than the overuse of BS in New York, but our survey confirms that its yield is low when the criteria are not fulfilled, even by using FCH PET/CT which is more expensive than BS.

Another interesting result of the present survey was the rather frequent indication of nuclear medicine examinations in the follow-up of therapy: 23% of the examinations. In this indication, FCH PET/CT has an important advantage over BS and fluoride(18F) PET/CT which are limited to the monitoring of bone lesions. Even for monitoring the metabolic response of bone metastases to therapy, FCH has the advantage to show the viable prostate cancer tissue while BS and fluoride(18F) PET/CT show the reaction of the normal cortical bone to the insult by the metastatic tissue. This difference in the mechanism of functional imaging explains the “bone flare phenomenon” observed on BS at the beginning of an active hormone therapy, which has even been proposed as a criterion to improve both sensitivity and specificity of BS in prostate cancer.^22^ In the evaluation of new therapeutic agents such as abiraterone, the effect of BS flare on the patient management and interpretation of results is clearly “confounding”.^23^ Nevertheless, NCCN recommends that patients treated with abiraterone or cabazitaxel with prednisone, must be monitored closely, in particular with BS, for evidence of progression.^2^ We foresee from the present survey that the application of FCH PET/CT to treatment monitoring will develop when this examination will become more widely available.

## Conclusions

In two PET centres of public hospitals of two EU member states, with a rather different context, the introduction of FCH PET/CT led to a rapid increase in its use, with a concomitant decrease in the number of nuclear medicine examinations devoted to the detection of bone metastases, but with an increase in the overall part of prostate cancer in nuclear medicine diagnostic practice: +24% in Paris and +100% in Ljubljana within one year. This shift for FCH PET/CT resulted in a greater proportion of positive examinations. Given the trend that was observed in our survey, it seems likely that FCH PET/CT will become the first line nuclear medicine examination in patients with prostate cancer disease. As prostate cancer is a frequent malignancy and the number of PET/CT machines is not sufficient in France and in Slovenia, more evidence-based criteria for its indication will be needed. It appears important that the referring physician mentions the initial Gleason score, the current PSA serum level, the recent evaluation of PSA level and all the therapeutic modalities. In our survey, the PSA levels and the Gleason score were available in only 43% of the prescriptions. According to our results, the criteria for referring patients at initial staging to BS appear to be suited for fluoride(18F) or FCH PET/CT. In contrast, the criteria for referring patients to BS in case of recurrent prostate cancer cannot apply to FCH PET/CT, which is more sensitive and specific and is also able to detect local recurrence and soft tissue invasion. The kinetics of variation of PSA levels may offer the best criteria in this context. FCH still lacks registration in the detection of prostate cancer in soft tissue as well as for therapy monitoring. It is unclear whether FDG will still have a role to play in the restaging and therapy monitoring of advanced forms of prostate cancer if FCH would be registered in those settings.

## Figures and Tables

**TABLE 1.** The two centres participating in the survey

**Centre**	**Type**	**BS may be completed with**	**FNa PET/CT**	**FDG PET/CT**	**FCH PET/CT**
France, Paris	Public university hospital	SPECT	since April 2008	since July 2004	since September 2004
Slovenia, Ljubljana	Public university hospital	SPECT/CT	Not available	since December 2009	since April 2010

BS = bone scintigraphy; FCH = fluorocholine(18F); FDG = fluorodeoxyglucose(18F); FNa = fluoride(18F); SPECT = single photon emission computed tomography

**TABLE 2.** Evolution of the number of examinations per quarter in Paris

	**1^st^ quarter**	**2^nd^ quarter**	**3^rd^ quarter**	**4^th^ quarter**	**5^th^ quarter**	**Total 1^st^–5^th^ quarters**
BS	20 (28%)	20 (22%)	24 (26%)	25 (28%)	14 (16%)	103
FNa PET/CT	35 (49%)	31 (34%)	39 (42%)	17 (19%)	24 (29%)	146
FDG PET/CT	8 (11%)	6 (7%)	6 (6%)	7 (8%)	7 (8%)	34
FCH PET/CT	8 (11%)	33 (37%)	24 (26%)	41 (46%)	39 (46%)	145
Total (100%)	71	90	93	90	84	428

BS = bone scintigraphy; FNa = fluoride(18F); FCH = fluorocholine(18F); FDG = fluorodeoxyglucose(18F)

**TABLE 3.** Evolution of the number of examinations per quarter in Ljubljana

	**1^st^ quarter**	**2^nd^ quarter**	**3^rd^ quarter**	**4^th^ quarter**	**5^th^ quarter**	**Total 1^st^–5^th^ quarters**
BS	6 (21%)	8 (19%)	6 (9%)	8 (13%)	1 (2%)	29
FDG PET/CT	0 (0%)	0 (0%)	1 (2%)	0 (0%)	0 (0%)	1
FCH PET/CT	23 (79%)	35 (81%)	58 (89%)	54 (87%)	60 (98%)	230
Total (100%)	29	43	65	62	61	260

BS = bone scintigraphy; FCH = fluorocholine(18F); FDG = fluorodeoxyglucose(18F)

**TABLE 4.** Indication of the nuclear medicine examination

**Indication**	**Initial**	**Follow-up**	**Restaging**	**Occult recurrence**	**All indications**
BS	60	26	22	24	132
FNa PET/CT	77	27	18	24	146
FDG PET/CT	2	17	14	2	35
FCH PET/CT	97	85	54	139	375
All examinations	236	155	108	189	688

BS = bone scintigraphy; FCH = fluorocholine(18F); FDG = fluorodeoxyglucose(18F); FNa = fluoride(18F); SPECT = single photon emission computed tomography

**TABLE 5.** Examination-based interpretation. For both “bone” and “soft tissue” “doubt” was only quoted if no focus evocative of malignancy was observed. % correspond to the frequency of this interpretation for each modality; since fluorodeoxyglucose(18F) (FDG) PET/CT and fluorocholine(18F) (FCH) PET/CT can detect foci in the prostatic bed, the soft tissue and the skeleton, the total is greater than 100%

**Interpretation**	**Number of examinations**	**Negative**	**Doubt bone**	**Bone metastasis**	**Prostate focus**	**Doubt soft tissue**	**Malignant soft tissue**
BS	132	65 (49%)	37 (28%)	30 (23%)	0	0	0
FNa PET/CT	146	79 (54%)	32 (22%)	35 (24%)	0	0	1 (1%)
FDG PET/CT	35	2 (3%)	1 (3%)	29 (83%)	1 (3%)	3 (9%)	8 (23%)
FCH PET/CT	375	52 (14%)	15 (4%)	86 (23%)	198 (53%)	21 (6%)	132 (35%)

BS = bone scintigraphy; FCH = fluorocholine(18F); FDG = fluorodeoxyglucose(18F); FNa = fluoride(18F)

## References

[1] Balogova S, Kobetz A, Huchet V, Michaud L, Kerrou K, Paycha F (2012). Évolution de la demande des examens de médecine nucléaire pour cancer de la prostate depuis l’enregistrement de la fluorocholine (18F): analyse sur deux ans à l’hôpital Tenon. Méd Nucl.

[2] Mohler JL, Armstrong AJ, Bahnson RR, Boston B, Busby JE, D’Amico AV (2012). Prostate cancer, Version 3.2012: featured updates to the NCCN guidelines. J Natl Compr Canc Netw.

[3] Even-Sapir E, Metser U, Mishani E, Lievshitz G, Lerman H, Leibovitch I (2006). The detection of bone metastases in patients with high-risk prostate cancer: ^99m^Tc-MDP planar bone scintigraphy, single- and multi-field-of-view SPECT, ^18^F-Fluoride PET, and ^18^F-Fluoride PET/CT. J Nucl Med.

[4] Langsteger W, Balogova S, Huchet V, Beheshti M, Paycha F, Egrot C (2011). Fluorocholine (18F) and sodium fluoride (18F) PET/CT in the detection of prostate cancer: prospective comparison of diagnostic performance determined by masked reading. Q J Nucl Med Mol Imaging.

[5] Talbot JN, Paycha F, Balogova S (2011). Diagnosis of bone metastasis: recent comparative studies of imaging modalities. Q J Nucl Med Mol Imaging.

[6] Hillner BE, Siegel BA, Shields AF, Liu D, Gareen IF, Hunt E (2008). Relationship between cancer type and impact of PET and PET/CT on intended management: findings of the National Oncologic PET Registry. J Nucl Med.

[7] Price DT, Coleman RE, Liao RP, Robertson CN, Polascik TJ, DeGrado TR (2002). Comparison of [18 F]fluorocholine and [18 F]fluorodeoxyglucose for positron emission tomography of androgen dependent and androgen independent prostate cancer. J Urol.

[8] McCarthy M, Siew T, Campbell A, Lenzo N, Spry N, Vivian J (2011). ^18^F-Fluoromethylcholine (FCH) PET imaging in patients with castration-resistant prostate cancer: prospective comparison with standard imaging. Eur J Nucl Med Mol Imaging.

[9] Schmid DT, John H, Zweifel R, Cservenyak T, Westera G, Goerres GW (2005). Fluorocholine PET/CT in patients with prostate cancer: initial experience. Radiology.

[10] Heinisch M, Dirisamer A, Loidl W, Stoiber F, Gruy B, Haim S (2006). Positron emission tomography/computed tomography with F-18-fluorocholine for restaging of prostate cancer patients: meaningful at PSA < 5 ng/ml?. Mol Imaging Biol.

[11] Cimitan M, Bortolus R, Morassut S, Canzonieri V, Garbeglio A, Baresic T (2006). [18F]fluorocholine PET/CT imaging for the detection of recurrent prostate cancer at PSA relapse: experience in 100 consecutive patients. Eur J Nucl Med Mol Imaging.

[12] Husarik DB, Miralbell R, Dubs M, John H, Giger OT, Gelet A (2008). Evaluation of (18F)-choline PET/CT for staging and restaging of prostate cancer. Eur J Nucl Med Mol Imaging.

[13] Pelosi E, Arena V, Skanjeti A, Pirro V, Douroukas A, Pupi A (2008). Role of whole-body 18F-choline PET/CT in disease detection in patients with biochemical relapse after radical treatment for prostate cancer. Radiol Med.

[14] Hodolič M (2011). Role of (18)F-choline PET/CT in evaluation of patients with prostate carcinoma. Radiol Oncol.

[15] Huchet V, Gutman F, Kerrou K, Cussenot O, Haab F, Doublet J (2007). Evaluation of PSA velocity as a selection criterion for FCH PET/CT in patients with biological recurrence of prostate cancer. Eur J Nucl Med Mol Imaging.

[16] Hodolic M, Maffione A, Fettich J, Gubina B, Cimitan M, Rubello D (2013). Metastatic prostate cancer proven by 18F-FCH PET/CT staging csan in patient with doubling time. Clin Nucl Med.

[17] Beheshti M, Imamovic L, Broinger G, Vali R, Waldenberger P, Stoiber F (2010). 18F choline PET/CT in the preoperative staging of prostate cancer in patients with intermediate or high risk of extracapsular disease: a prospective study of 130 patients. Radiology.

[18] Poulsen MH, Bouchelouche K, Høilund-Carlsen PF, Petersen H, Gerke O, Steffansen (2012). [18F]fluoromethylcholine (FCH) positron emission tomography/computed tomography (PET/CT) for lymph node staging of prostate cancer: a prospective study of 210 patients. BJU Int.

[19] Kjölhede H, Ahlgren G, Almquist H, Liedberg F, Lyttkens K, Ohlsson T (2012). Combined 18F-fluorocholine and 18F-fluoride positron emission tomography/computed tomography imaging for staging of high-risk prostate cancer. BJU Int.

[20] Briganti A, Passoni N, Ferrari M, Capitanio U, Suardi N, Gallina A (2010). When to perform bone scan in patients with newly diagnosed prostate cancer: external validation of the currently available guidelines and proposal of a novel risk stratification tool. Eur Urol.

[21] Lavery HJ, Brajtbord JS, Levinson AW, Nabizada-Pace F, Pollard ME, Samadi DB (2011). Unnecessary imaging for the staging of low-risk prostate cancer is common. Urology.

[22] Cook GJ, Venkitaraman R, Sohaib AS, Lewington VJ, Chua SC, Huddart RA (2011). The diagnostic utility of the flare phenomenon on bone scintigraphy in staging prostate cancer. Eur J Nucl Med Mol Imaging.

[23] Ryan CJ, Shah S, Efstathiou E, Smith MR, Taplin ME, Bubley GJ (2011). Phase II study of abiraterone acetate in chemotherapy-naive metastatic castration-resistant prostate cancer displaying bone flare discordant with serologic response. Clin Cancer Res.

